# Risk reduction of severe outcomes in vaccinated COVID-19 cases: an analysis of surveillance data from Estonia, Ireland, Luxembourg and Slovakia, January to November 2021

**DOI:** 10.2807/1560-7917.ES.2022.27.7.2200060

**Published:** 2022-02-17

**Authors:** Gaetano Marrone, Nathalie Nicolay, Nick Bundle, Tommi Karki, Gianfranco Spiteri, Heleene Suija, Kerstin-Gertrud Kärblane, Joël Mossong, Anne Vergison, Maria Avdicova, Adriana Mecochova, Gillian Cullen, Piaras O’Lorcain, Lucia Pastore Celentano, Tarik Derrough, Julien Beauté

**Affiliations:** 1 European Centre for Disease Prevention and Control (ECDC), Solna, Sweden; 2 Republic of Estonia Health Board, Tallinn, Estonia; 3 Health Directorate, Luxembourg City, Luxembourg; 4 Regional Public Health Authority, Banska Bystrica, Slovakia; 5 Public Health Authority of the Slovak Republic, Bratslava, Slovakia; 6 Health Protection Surveillance Centre, Dublin, Ireland

**Keywords:** COVID-19, surveillance, vaccination, vaccine effectiveness, Europe Union, hospitalization, death

## Abstract

Despite high COVID-19 vaccine coverage in the EU/EEA, there are increasing reports of SARS-CoV-2 infections and hospitalisations in vaccinated individuals. Using surveillance data from Estonia, Ireland, Luxembourg and Slovakia (January–November 2021), we estimated risk reduction of severe outcomes in vaccinated cases. Increasing age remains the most important driver of severity, and vaccination significantly reduces risk in all ages for hospitalisation (adjusted relative risk (aRR): 0.32; 95% confidence interval (CI): 0.26–0.39) and death (aRR: 0.20; 95% CI: 0.13–0.29).

In the European Union/European Economic Area (EU/EEA) as at 7 November 2021, ca 76% (country range: 27–92) of the adult population aged 18 years and older had received the complete primary vaccination series [1] against coronavirus disease (COVID-19), most with two doses of Comirnaty (BNT162b2 mRNA, BioNTech-Pfizer, Mainz, Germany/New York, United States (US)) (70% of doses administered) [2]. While the rollout of the COVID-19 vaccines has been an effective public health measure in preventing further disease burden [3,4], the increasing number of breakthrough infections with severe acute respiratory syndrome coronavirus 2 (SARS-CoV-2) reported among fully vaccinated individuals has raised concerns about vaccine effectiveness, especially among older age groups (≥ 60 years) [5]. We estimated the risk reduction of severe outcomes in fully vaccinated COVID-19 cases using surveillance data reported to the European Centre for Disease Prevention and Control (ECDC).

## Surveillance of COVID-19 cases in the EU/EEA

ECDC and the World Health Organization Regional Office for Europe (WHO/Europe) jointly coordinate COVID-19 surveillance in Europe. Since 27 January 2020, countries have reported weekly data on COVID-19 cases to The European Surveillance System (TESSy) [6]. The variables included in the case-base reporting are age, sex, medical preconditions, hospital admission and death. From 12 March 2021, vaccination status, vaccination date, number of doses received and vaccine product were also registered in TESSy.

## Quality of EU/EEA COVID-19 surveillance data

For the purpose of this retrospective cohort study, we considered cases to be all adult individuals aged 18 years and older with laboratory-confirmed SARS-CoV-2 [7] as reported to TESSy by EU/EEA countries since 1 January 2021 when vaccination rollout started. We included countries who reported a minimum of 75% of their cases with information on vaccination status and date of vaccination for at least 1 month. We defined fully vaccinated as having received the complete primary vaccination series, i.e. one dose (COVID-19 Vaccine Janssen, Janssen-Cilag International NV, Beerse, Belgium) or two doses (Comirnaty/BNT162b2 mRNA, BioNTech-Pfizer, Mainz, Germany/New York, United States (US); Spikevax/mRNA-1273, Moderna, Cambridge, US; Vaxzevria/ChAdOx1 nCoV-19, Oxford-AstraZeneca, Cambridge, United Kingdom) of COVID-19 vaccine as per official recommendations [1].

During weeks 1 to 45 2021, eight of the 19 countries reporting case-based data reported cases with information on vaccination status (Austria, Estonia, Ireland, Luxembourg, the Netherlands, Poland, Portugal, and Slovakia). Of these countries, four had completeness for the variables of vaccination status and vaccination date above 75% for at least 1 month (Estonia: August–October, Ireland: April–November, Luxembourg: March–October and Slovakia: January–November).

## Estimating risk reduction

We described several characteristics of reported COVID-19 cases, including sex, age (in four groups), underlying medical conditions, hospital admission, and outcome (alive/dead) by COVID-19 vaccination status.

We ran negative binomial regression models with a standard error robust to country-related intra-cluster correlation, to calculate the relative risks (RR) and the 95% confidence interval (CI) of hospitalisation and death for fully vaccinated individuals compared with those unvaccinated, adjusting by sex, age group, underlying medical conditions and reporting country.

We further stratified the regression analysis by age group and by two study periods according to the circulation of the SARS-CoV-2 Delta variant (Phylogenetic Assignment of Named Global Outbreak LINeages (Pangolin) designation B.1.617.2): the pre-Delta study period (Delta was not dominant) and the Delta study period (Delta was dominant). We excluded the weeks of co-circulation, which varied slightly for each country (weeks 23–28 for Estonia, 22–28 for Ireland, 19–25 for Luxembourg, 25–28 for Slovakia). We have also further categorised the fully vaccinated individuals as having completed the primary vaccination series less than or more than 6 months before COVID-19 onset date.

## Characteristics of COVID-19 cases in four EU countries by vaccination status

During weeks 1 to 45 2021, Estonia, Ireland, Luxembourg and Slovakia (total pop. 12.4 million, i.e. 2.7% of the total EU-27 population) reported 574,026 cases with complete information on vaccination status and date of vaccine administration. Of these, 488,210 (85.1%) were unvaccinated, 75,598 (13.2%) fully vaccinated and 10,218 (1.8%) partially vaccinated. We excluded the latter from the analysis since the maximum protective effect would only be expected 14 days after completion of the primary vaccination series. Of the 563,808 remaining cases with known vaccination status, 431,137 (76.5%) reported information on hospitalisation status, of which 19,652 (4.6% of the known cases) were admitted to hospital (Table 1). Of the 562,289 (99.7%) cases reported with information on outcome, 8,590 (1.5%) cases died. Compared with those unvaccinated, individuals who had received the complete primary vaccination series were older (median age: 47 (IQR: 37–61) vs 42 (IQR: 31–54) years) but less likely to be reported with a severe outcome (2.3% vs 3.7% for hospitalisation and 0.4% vs 1.7% for death) (Table 1). The proportion of cases reported with a severe outcome increased with age reaching 35.3% and 21.2% for those aged 80 years and above for hospitalisation and death, respectively (Table 2).

**Table 1:** Characteristics of COVID-19 cases by vaccination status reported to The European Surveillance System (TESSy), Estonia, Ireland, Luxembourg, and Slovakia, weeks 1–45 2021 (n = 563,808)

Characteristics	Total COVID-19 cases (n = 563,808)		Unvaccinated (n = 488,210)		Fully vaccinated (n = 75,598)	
	n	%	n	%	n	%
Sex						
Women	291,211	51.7	249,188	51.0	42,023	55.6
Men	272,597	48.3	239,022	49.0	33,575	44.4
Age at diagnosis (years)						
18–49	366,388	65.0	324,904	66.6	41,484	54.9
50–64	130,262	23.1	110,131	22.6	20,131	26.6
65–79	55,036	9.8	43,133	8.8	11,903	15.7
≥ 80	12,122	2.1	10,042	2.1	2,080	2.8
Underlying medical condition(s)						
No	8,854	1.6	7,304	1.5	1,550	2.1
Yes	7,146	1.3	6,322	1.3	824	1.1
Other	221,168	39.2	195,579	40.1	25,589	33.8
Unknown	326,640	57.9	279,005	57.1	47,635	63.0
Admitted to hospital						
No	411,485	73.0	346,618	71.0	64,867	85.8
Yes	19,652	3.5	17,908	3.7	1,744	2.3
Unknown	132,671	23.5	123,684	25.3	8,987	11.9
Outcome						
Alive	553,699	98.2	479,185	98.1	74,514	98.6
Dead	8,590	1.5	8,260	1.7	330	0.4
Unknown	1,519	0.3	765	0.2	754	1.0

COVID-19: coronavirus disease.

Fully vaccinated cases received the complete primary vaccination series, i.e. one dose (COVID-19 Vaccine Janssen, Janssen-Cilag International NV, Beerse, Belgium) or two doses (Comirnaty/BNT162b2 mRNA, BioNTech-Pfizer, Mainz, Germany/New York, United States; Spikevax/mRNA-1273, Moderna, Cambridge, US; Vaxzevria/ChAdOx1 nCoV-19, Oxford-AstraZeneca, Cambridge, United Kingdom) of COVID-19 vaccine as per official recommendations.

**Table 2:** Characteristics and relative risk of hospitalisations and deaths among COVID-19 cases reported to The European Surveillance System (TESSy), Estonia, Ireland, Luxembourg and Slovakia, weeks 1–45 2021

Characteristics	All records	Hospitalisations	%	aRR^a^	95% CI	All outcomes	Deaths	%	aRR^a^	95% CI
Total	431,137	19,652	4.6	NA		562,289	8,590	1.5	NA	
Sex										
Women	225,683	9,715	4.3	Ref.		290,468	3,992	1.4	Ref.	
Men	205,454	9,937	4.8	1.30	1.18–1.44	271,821	4,598	1.7	1.63	1.61–1.65
Age at diagnosis (years)										
18–49	278,364	3,675	1.3	Ref.		365,379	337	0.1	Ref.	
50–64	99,419	4,898	4.9	3.94	3.52–4.40	129,965	1,638	1.3	13.31	11.17–15.84
65–79	43,265	7,521	17.4	13.94	11.57–16.79	54,858	4,050	7.4	77.28	63.28–94.39
≥ 80	10,089	3,558	35.3	29.09	26.23–32.27	12,087	2,565	21.2	225.70	174.83–291.37
Underlying medical condition(s)										
No	8,854	197	2.2	Ref.		8,854	5	0.1	Ref.	
Yes	7,134	2,057	28.8	4.27	3.94–4.62	7,146	1,006	14.1	23.07	20.77–25.63
Other	218,603	11,162	5.1	1.59	1.49–1.67	221,168	4,690	2.1	9.00	8.20–9.90
Unknown	196,546	6,236	3.2	0.92	0.82–1.03	221,168	2,889	0.9	4.79	4.40–5.22
Vaccination status										
Unvaccinated	364,526	17,908	4.9	Ref.		487,445	8,260	1.7	Ref.	
Fully vaccinated	66,611	1,744	2.6	0.32	0.26–0.39	74,844	330	0.4	0.20	0.13–0.29

aRR: adjusted relative risk; CI: confidence interval; COVID-19: coronavirus disease; NA: not applicable; Ref: reference.

^a^ The aRR are obtained by a negative binomial regression models with a standard error robust to country-related intra-cluster correlation.

Fully vaccinated cases received the complete primary vaccination series, i.e. one dose (COVID-19 Vaccine Janssen, Janssen-Cilag International NV, Beerse, Belgium) or two doses (Comirnaty/BNT162b2 mRNA, BioNTech-Pfizer, Mainz, Germany/New York, United States; Spikevax/mRNA-1273, Moderna, Cambridge, US; Vaxzevria/ChAdOx1 nCoV-19, Oxford-AstraZeneca, Cambridge, United Kingdom) of COVID-19 vaccine as per official recommendations.

## Risk reduction of severe outcomes in vaccinated COVID-19 cases

Over the study period and in the four countries included in the analysis, the COVID-19 hospital admissions rate was lower in fully vaccinated individuals compare to unvaccinated (Figure). Being 80 years of age and older was associated with an adjusted RR of hospitalisation and death of 29.1 (95% CI: 26.2–32.3) and 225.7 (95% CI: 174.8–291.4), respectively (Table 2). In addition to age, being male and having an underlying medical condition were also associated with a higher risk of both hospitalisation and death. Overall, full vaccination was associated with a reduced risk of hospitalisation (aRR = 0.32; 95% CI: 0.26–0.39) and death (aRR = 0.20; 95% CI: 0.13–0.29) respectively. The risk reduction of severe outcomes associated with full vaccination was significant in all age groups but decreased with increasing age (Table 3).

**Table 3:** Adjusted relative risk of hospitalisations and deaths in fully vaccinated versus unvaccinated COVID-19 cases by age group, Estonia, Ireland, Luxembourg, Slovakia, as of weeks 01–45 2021

Age (years)	Hospitalisations in fully vaccinated cases^a^		Deaths in fully vaccinated cases^a^	
	aRR^b^	95% CI	aRR^b^	95% CI
18–49	0.37	0.32–0.44	0.09	0.02–0.48
50–64	0.25	0.22–0.28	0.14	0.05–0.38
65–79	0.33	0.28–0.40	0.16	0.14–0.18
≥ 80	0.58	0.51–0.66	0.28	0.24–0.34

aRR: adjusted relative risk; CI: confidence interval; COVID-19: coronavirus disease.

^a^ Reference group: unvaccinated.

^b^ Model adjusted for sex, underlying medical conditions and reporting country. The aRR are obtained by a negative binomial regression models with a standard error robust to country-related intra-cluster correlation.

Fully vaccinated cases received the complete primary vaccination series, i.e. one dose (COVID-19 Vaccine Janssen, Janssen-Cilag International NV, Beerse, Belgium) or two doses (Comirnaty/BNT162b2 mRNA, BioNTech-Pfizer, Mainz, Germany/New York, United States; Spikevax/mRNA-1273, Moderna, Cambridge, US; Vaxzevria/ChAdOx1 nCoV-19, Oxford-AstraZeneca, Cambridge, United Kingdom) of COVID-19 vaccine as per official recommendations.

**Figure:**
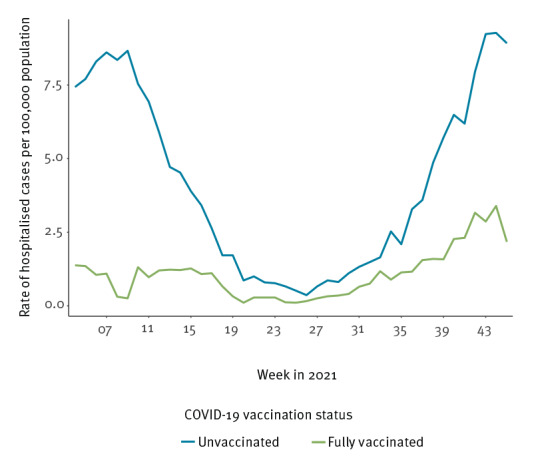
Rate of hospitalisation of COVID-19 cases by vaccination status reported to The European Surveillance System (TESSy), Ireland, Luxembourg and Slovakia, weeks 1–45 2021

COVID-19: coronavirus disease.

Estonia only reported a subset of their cases in case-based format, i.e. with information on both vaccination status and admission to the hospital, and therefore was not included.

Fully vaccinated cases received the complete primary vaccination series, i.e. one dose (COVID-19 Vaccine Janssen, Janssen-Cilag International NV, Beerse, Belgium) or two doses (Comirnaty/BNT162b2 mRNA, BioNTech-Pfizer, Mainz, Germany/New York, United States; Spikevax/mRNA-1273, Moderna, Cambridge, US; Vaxzevria/ChAdOx1 nCoV-19, Oxford-AstraZeneca, Cambridge, United Kingdom) of COVID-19 vaccine as per official recommendations.

Similar results were observed when stratifying the analysis by Delta variant circulating period, by completion of a two-dose vaccination series for less or more than 6 months before the onset date, and when the regression models were further adjusted by month of COVID-19 onset (data not shown).

## Ethical statement

This study was based on national surveillance data submitted to ECDC. Therefore, written informed consent from the patients was not required due to the anonymous nature of the data.

## Discussion

We observed a sizeable proportion of fully vaccinated COVID-19 cases reported with severe COVID-19 disease, especially in older age groups. This is not unexpected given the high level of full vaccination uptake, which ranged from between 60.3% (Slovakia) and 100% (Ireland) in those aged 80 years and above, as at week 45 2021 [8]. COVID-19 vaccine breakthrough is expected for several reasons. Firstly, the COVID-19 vaccines are not 100% effective [9]. Secondly, there is evidence that vaccine-acquired immunity wanes over time, especially in immunocompromised and older individuals [10]. Lastly, SARS-CoV-2 continues to evolve and new variants such as the highly contagious Delta have shown potential immune evasive properties which could further contribute to a slightly reduced vaccine effectiveness. A recent systematic review suggested that there could be a 10 to 20% reduction of vaccine effectiveness against infection with the Delta variant, although protection against severe COVID-19 is still maintained [11]. Yet, it is important to monitor and characterise infections in fully vaccinated individuals, especially those with severe outcomes, as this would allow a better understanding of the pandemic and help inform the possible need for revisions of vaccination strategies. Our findings confirm that COVID-19 in older age groups remains associated with high risk of severe outcomes, even if the vaccine reduces this risk significantly over both the pre-Delta and Delta circulating periods. The benefits of a third dose of the vaccine should be assessed in this context.

Our analysis has limitations, including possible reporting bias with severe cases more likely to be reported to ECDC. This would lead to an overestimation of the hospitalisation risk, especially in vaccinated individuals. Similarly, vaccinated cases may be less likely to be tested, which would lead to an overestimation of risk of severe outcome in this group. We therefore think that our estimates are conservative. Although testing strategies and non-pharmaceutical measures changed over time and across countries, we were unable to consider them here in detail. However, those changes are unlikely to have impacted our findings since they are not expected to influence case outcomes by vaccination status. During the study period, no country restricted testing according to vaccination status and testing rates were high, suggesting that access to testing was adequate. In addition, our estimates were adjusted by country. Lastly, data quality hindered the inclusion of additional countries.

## Conclusion

Our data showed that the occurrence of severe outcomes is less likely among fully vaccinated COVID-19 cases. However, the risk of severe outcomes remains driven by age and is particularly high for older age groups despite vaccination status.
